# Correction: Saadeh et al. Recent Advances in the Synthesis and Biological Activity of 8-Hydroxyquinolines. *Molecules* 2020, *25*, 4321

**DOI:** 10.3390/molecules27134306

**Published:** 2022-07-05

**Authors:** Haythem A. Saadeh, Kamal A. Sweidan, Mohammad S. Mubarak

**Affiliations:** 1 Department of Chemistry, College of Science, United Arab Emirates University, Al Ain P.O. Box 15551, United Arab Emirates; h.saadeh@uaeu.ac.ae; 2 Department of Chemistry, School of Science, The University of Jordan, Amman 11942, Jordan; k.sweidan@ju.edu.jo

The author wishes to make the following correction to this paper [1]. Due to mislabeling, replace:

**Scheme 7:**
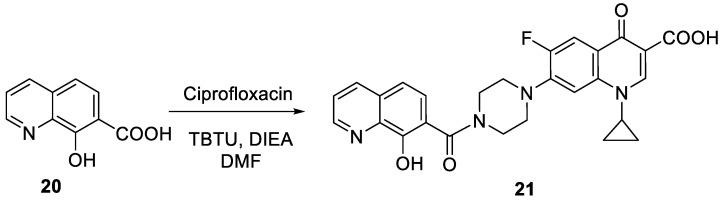
Preparation of 8-hydroxyquinoline-ciprofloxacin hybrid **21**.

with

**Scheme 7:**
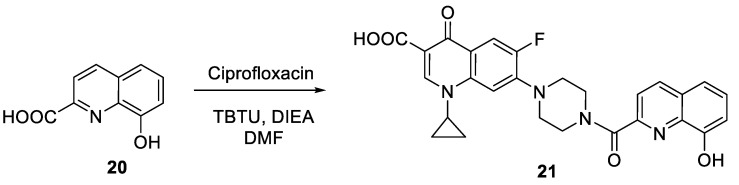
Preparation of 8-hydroxyquinoline-ciprofloxacin hybrid **21**.
